# The recruitment legacy of COVID-19

**DOI:** 10.1192/bjb.2020.63

**Published:** 2020-08

**Authors:** Rajaei K. Sharma, Bhupinder Sharma, Harriet L. Ogle

**Affiliations:** Medical Student, University of Exeter, UK; email: rajaei.sharma@nhs.net; Consultant, Royal Marsden Hospital, UK NICE Expert Advisor Centre for Guidelines Development, UK; Medical Student, University of Exeter, UK

With increasing thought being put into COVID-19 and the implications for psychiatric care in the months and years to come, it would be remiss to not consider the potential impact this could have on recruitment to the specialty.

Recruitment in psychiatry is a national, if not global problem, and has been for some time. Although frequently highlighted, there has been limited work clarifying the reasons behind the problem, which in turn has led to muted and potentially ineffective responses. This could all now be amplified by the COVID-19 crisis.

We know that foundation doctors already have very low exposure to psychiatry placements and teaching; the proportion of psychiatry teaching time has been found to be as low as 2.3% relative to surgical and medical specialties seeing 44.1%. We also know that there is a significant correlation between trainees having a foundation placement in psychiatry and going on to apply for specialty training.^1^ This has been replicated in targeted studies with a surprisingly high 45% of psychiatric specialists found to not have even considered the specialty before their foundation years.^2^

So, it is not an understatement that the current foundation year trainees are to be drastically affected. Not only have rotations been paused, but huge numbers have been redeployed. Already, the invaluable experiences of an entire cohort have been curtailed, with more likely to follow. Additionally, national exams have been cancelled; the impact of this and the choices made thereafter may even lead to very immediate-term shortages and unknown ramifications.

This is before we even consider those that will shortly follow them through, the medical students. The proportionally enormous amount of time lost directly affects many of the clearly identified factors attracting students to the field such as placement and elective exposure.^3^ There have recently been some targeted strategies implemented by the Royal College of Psychiatry to try and improve recruitment – especially the ‘Choose Psychiatry’ campaign – these outreach programmes might serve to be a much-needed lifeline more than ever before. Psychiatric societies within universities might be the great untapped resource that prevail the shutdown and educational restrictions.^4^ Could they, in turn, be a vital commodity?

There is a growing recognition that although it may appear to be minutiae relative to the scale of a global pandemic, COVID-19 could have a profound effect on the career progressions of a generation of doctors.^5^ Only by addressing this early and actively can we mitigate potential disaster for years to come.
